# Autism, Therapy and COVID-19

**DOI:** 10.3390/pediatric13010005

**Published:** 2021-01-05

**Authors:** Luana Sergi, Emanuele Mingione, Maria Carla Ricci, Antonella Cavallaro, Ferdinando Russo, Giulio Corrivetti, Francesca Felicia Operto, Alessandro Frolli

**Affiliations:** 1 ASL (Local Health Authority) of Caserta, 81100 Caserta, Italy; luana.sergi@aslcaserta.it (L.S.); emauele.mingione@aslcaserta.it (E.M.); ferdinando.russo@aslcaserta.it (F.R.); 2 Disability Research Centre, University of International Studies in Rome, 00147 Roma, Italy; mariacarla.ricci1@gmail.com (M.C.R.); a-cavallaro@live.it (A.C.); 3 Department of Mental Health of ASL (Local Health Company) of Salerno, 81100 Caserta, Italy; corrivetti@gmail.com; 4 Child and Adolescent Neuropsychiatry Unit, Department of Medicine, Surgery and Dentistry, University of Salerno, 84084 Fisciano, Italy; opertofrancesca@gmail.com

**Keywords:** ABA, autism, quarantine, COVID-19, children, communication, stereotypes, behaviors

## Abstract

While numerous treatments for ASD are available, intervention based on the principles and procedures of Applied Behavior Analysis (ABA) has garnered substantial scientific support. In this study we evaluated the effects of the lockdown during the COVID-19 pandemic outbreak, followed by quarantine provisions and during the three months after the resumption of activities. The study was conducted on a group of children taking part on a ABA-based intervention funded by the Local Health Authority (ASL) of the province of Caserta. In this study we considered a sample of 88 children who had been diagnosed with Autism Spectrum Disorder, aged between 18 and 30 months. The following inclusion criteria were observed: age at the time of diagnosis less than 30 months, absence of other neurological, genetic, or sensorineural pathologies, and severity level 1 measured by symptoms evaluation based on the ADOS 2 module T (used for diagnosis). During the lockdown children experienced improvements in communication, socialization, and personal autonomy. During the three months after the ABA treatment, the acquired skills were maintained but no significant improvement was demonstrated. In this study, we describe how parent training was significant in avoiding delays in the generalization of socially significant behaviors, following the drastic interruption of the treatment in this group of children.

## 1. Introduction

Autism Spectrum Disorder (ASD) is a neurodevelopmental disorder described in Diagnostic and Statistical Manual of Mental Disorders [1], characterized by a pervasive delay in language development and socialization, and the presence of stereotyped, repetitive behaviors or non-functional interests. Restricted and repetitive behaviors seen in children with ASD include repetitive body language stereotypes (e.g., clapping hands, walking on toes, flickering, etc.) limited use of objects (object alignment, matching by color and shape, attention focused on the same game for a long amount of time etc.), sensory alterations (hypo- or hypersensitivity to sounds, smells, lights, etc.) and/or active engagement in atypical rituals (sitting in the same place, using the same cutlery, etc.). Among the core deficits of this communicative-social disorder, joint attention is particularly relevant: this represents the ability to share a common focus of interest with another and in children with ASD it is compromised both in initiative and in response [2]. Children with typical development tend to engage with interesting objects, observing and pointing at them with curiosity and then seeking attention from their parents to share the experience; children with ASD are instead less likely to engage in such forms of social attention, as they are more inclined not to show interest, or share initiatives proposed by the parent or peers. In fact, joint attention represents a signal of the capacity for social reference and its deficit expresses a broader impairment in sharing experiences and in the social drive. Manifestations of ASD symptoms vary greatly, leading to large clinical heterogeneity with a wide expression of symptoms. Interestingly, most children with ASD do not have intellectual disabilities and only a small percentage (10%–15%) is associated with cognitive impairment [3]. The latest prediction on the prevalence of AS, reveals a figure of 1 in 68 for the 2014 data, and 1 in 49 for the data published in 2018 by the Centers for Disease Control and Prevention [4], with a clear male prevalence (4.5:1). At an international level, the main priority is to initiate an intensive intervention to facilitate the development of socially significant behaviors from the post-early diagnosis stages. There are numerous treatments for ASD, but intervention based on the principles and procedures of Applied Behavior Analysis (ABA) has garnered substantial scientific support. This approach was first applied to treat autism in the 1960s by Ivar Lovaas who developed the first entirely ABA-based treatment, commonly referred to as Early and Intensive Behavioral Intervention (EIBI) [5]. Since then, ABA has been the subject of numerous studies published in peer-reviewed journals [6] and has inspired numerous evidence-based treatment models not only for autism but also for other neurodevelopmental disorders, such as Incidental Teaching [7], Pivotal Response Training (PRT) [8,9,10,11] and Early Start Denver Model (ESDM) [12,13,14,15] to name a few. There is a substantial body of research supporting ABA for children with autism, which has led several independent entities to recognize ABA and approve its use for children with ASD, including from the United States Surgeon General [16], the New York State Department of Health [17], and the National Academy of Sciences [18]. The impact of such confirmation is evidenced by changes in public policies such as formal state funding awarded for autism treatment and state-level legislative decisions requiring insurance coverage for ABA treatment (e.g., “Steven’s Law”, Arizona House Bill 2487). Additionally, in Italy, public health bodies are funding research in line with the Istituto Superiore di Sanità guidelines. In the province of Caserta, the Local Health Authority has operated for some years an ABA-based qualification program for a maximum of 15 h of weekly treatment that can be arranged at home, school or rehabilitation center. Monthly supervision is also a part of the program, by a figure who is Board Certified Behavior Analyst (BCBA) as indicated by the international guidelines of the Behavior Analyst Certification Board (BACB) on treatments for Autism Spectrum Disorder. In this study we evaluated the effects of the lockdown during the COVID-19 pandemic outbreak, followed by quarantine provisions, and the three months after the resumption of activities. The study was conducted on a group of children aged between 20 and 30 months taking part in an ABA-based intervention funded by the Local Health Authority (ASL) of the province of Caserta. The study was performed to evaluate the stabilization and generalization characteristics of an ABA-based intervention in children affected with early ASD. In this work we investigated the maintenance of previously acquired skills and the progression of socially significant behaviour during the treatment’s suspension period and during the three months after the resumption of treatment post-suspension. Our research also considers the behavioral problems that occurred during the suspension and resumption of treatment, particularly regarding stereotyped behaviour and rituals.

## 2. Materials and Methods

### 2.1. Participants

In this study we considered a sample of 88 children diagnosed with Autism Spectrum Disorder, aged between 20 and 30 months (mean age 23 months, SD 0.41). The inclusion criteria were as follows: (a) age less than 30 months at the time of diagnosis; (b) absence of other neurological, genetic or sensorineural pathologies; (c) severity level 1 (total average score and standard deviation) (PT 6—SD 0.31) obtained through symptoms evaluation by the ADOS 2 module T (diagnostic test). ABA treatment of 15 h per week was provided to children diagnosed with Autism Spectrum Disorder as part of a specific project. The treatment could take place in different settings, such as the children’s’ home, school or an affiliated facility, with monthly supervision by a certified BCBA practitioner. All subjects were treated for six months, before the extraordinary closure of services and an interruption of treatment occurred during the COVID 19 outbreak (lockdown period: from mid-March to mid-May 2020). Age distribution, diagnosis, severity of diagnosis, absence of co-morbidities and duration of pre-discontinuation of the treatment were sufficiently homogeneous among the sample. The absence of psychopathological correlates in the parents, the middle-high socio-cultural class of the families, and parental age represented another element of homogeneity in the sample. In particular, the mean maternal age was 29 years (SD 0.41) and the mean paternal age was 33.4 years (SD 0.67). The data were collected at the Center for the Monitoring of Autism Spectrum Disorders, part of the local health authority (ASL) of Caserta, in collaboration with the University of International Studies of Rome (UNINT) and with the mental health department of the ASL of Salerno.

### 2.2. Procedures and Tasks

The protocol implemented consisted of the following tests: ADOS 2—Toddler Module (Autism Diagnostic Observation Schedule) [19], ADI-R (Autism Diagnostic Interview-Revised) [20] and VABS II (Vineland Adaptive Behavior Scales II) [21].

#### 2.2.1. ADOS 2—Toddler Module

This is a standardized semi-structured observation aiming to evaluate communication and mutual interaction. The child is enrolled in 11 activities, with a duration range of between 30 and 45 min, under the observation of a caregiver. The Toddler Module was developed for children up to 30 months of age, who can walk autonomously, with limited language and a chronological and non-verbal age of at least 12 months. The Toddler Module follows a structure similar to that of the other modules: it must be conducted in a room specifically for children, and, during the activity, parents must always be present. Both cause-effect toys and representative and imaginative toys are included. The Toddler Module allows the evaluation of the child’s ability to behave appropriately when particular situations arise (fun shared with the adult, appropriate requests, search for others).

#### 2.2.2. ADI-R (Autism Diagnostic Interview-Revised)

This is a semi-structured interview, aimed at caregivers and composed of 93 items. The interview addresses current adopted behaviors of children between four and five years of age to identify the following: (i) anomalies in mutual social interaction, (ii) qualitative anomalies in communication, (iii) patterns of repetitive behaviour, and (iv) stereotyped restricted behaviour. It focuses on the systematic and standardized observation of behaviors that are rarely found in non-clinical subjects, and mainly on the three areas of functioning, namely: language and communication, mutual social interaction, stereotyped behaviour and restricted interests. ADI-R can be performed at various ages for diagnosis or intervention, following the structure of an interview protocol and five algorithms. If the intent of the evaluation is to formulate a formal diagnosis, one of the two diagnostic algorithms (2–3–11 years; 4 years or more) is adopted. If, on the other hand, the intent is to plan therapy or an educational project, the algorithm will be chosen according to the adopted behavior (3–11 years; 4–11 years; 10 years and over).

#### 2.2.3. Vineland II (Vineland Adaptive Behavior Scales II, VABS-II)

This is a semi-structured interview to evaluate Adaptive Behaviour (AB). It includes activities carried out by the individual to meet expectations of personal autonomy and social responsibility typical for people of the same age and cultural context. Specifically, semi-structured interviews measure AB in the subscales of Communication, Personal Autonomies, Socialization, and Motor Skills. In line with the diagnostic criteria of DSM-5, the interview allows us to establish the level of severity of the disorder.

#### 2.2.4. Questionnaire

The questionnaire included five closed questions on the following behavioral aspects: hyperactivity, attention, sharing aspects and search of the other, rituals, and stereotypes. The answers were provided based on a 5-point Likert scale which ranged from a minimum value of 1 to a maximum value of 5, to measure the intensity of the behaviour. The questions postulated to the parents were based on the corresponding items of the ADOS-2 Module T. For instance, question 4 was formulated as follows: “Does your child demonstrate atypical behaviors, such as sitting in the same place, looking for food of the same color, wearing the same garment, etc.”. In particular, the first question corresponded to items E1, the second to items B13, the third to items B11, the fourth to items B15, and the fifth to items D5. The questionnaire administered at the time of the diagnosis showed in all 88 subjects a significant correlation between question and the corresponding item of ADOS 2 Module T.

## 3. Procedures

The children included in this study had received a diagnosis of Autism Spectrum Disorder meeting the criteria of DSM 5 [1]. Clinical evaluation was completed through the administration of the following tests: ADOS 2—Toddler Module (Autism Diagnostic Observation Schedule) and ADI-R (Autism Diagnostic Interview-Revised). These tests were used exclusively at the time of diagnosis. With the introduction of the ABA treatment, an assessment of the child’s adaptive functioning and autonomy was carried out every three months, through the administration of VABS II with the parents. The administration took place at the beginning of the lockdown and was performed over the phone (March 2020). A subsequent administration was performed at the end of the lockdown (June 2020), and another followed after three months from the resumption of treatment (September 2020). We built an ad hoc questionnaire, completed over the phone by the parents, at the beginning of the lockdown, to assess the behavioral aspects of the children, then the same questionnaire was repeated at the end of the lockdown and three months after the resumption of treatment. Data obtained from the various measurements were collected to investigate improvements or worsening of the behavioral aspects of the children taking part in the project. The investigation was carried out to verify the robustness of the results and the generalization of socially significant behaviors during the period of interruption and resumption of treatment. Data collection was performed after the parents’ telephonic acceptance of the consent from for the processing of sensitive data. The acceptance of the treatment (15 h per week and a monthly supervision) was given at the start of the treatment proposed by the ASL (public health authority).

## 4. Methods

Data analysis was performed using the SPSS 26.0 [22] statistical survey software. The significance of the statistical results was accepted at the 5% level (α < 0.05). The comparison of group averages was carried out through a variance analysis test (Analysis of Variance—ANOVA), a parametric test that analyses two or more data groups by comparing internal variability between the groups. The relationship between these variances follows Fisher’s F distribution, to examine the hypotheses for the significance of the difference between the variability due to treatment and the residual one. In this study, we performed an ANOVA to compare the scores that emerged from the different measurements, carried out at the time of lockdown (T0), at the end of lockdown (T1) and three months after the resumption of ABA treatment (T2), respectively.

## 5. Results

**Figure 1:**
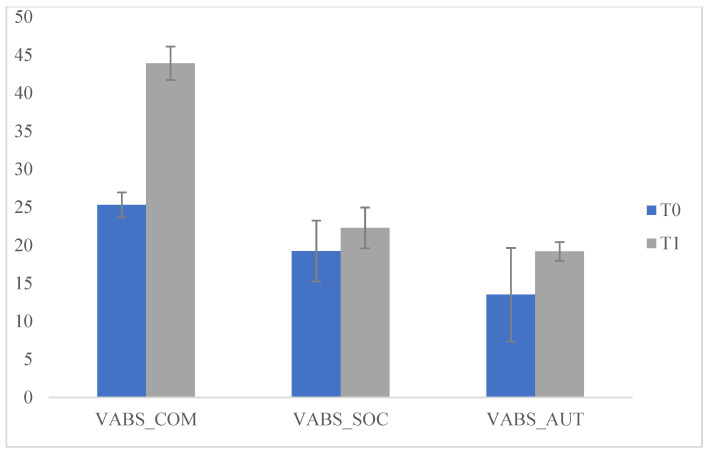
Comparisons of Vineland Adaptive Behavior Scales (VABS), T0 and T1.

**Figure 2:**
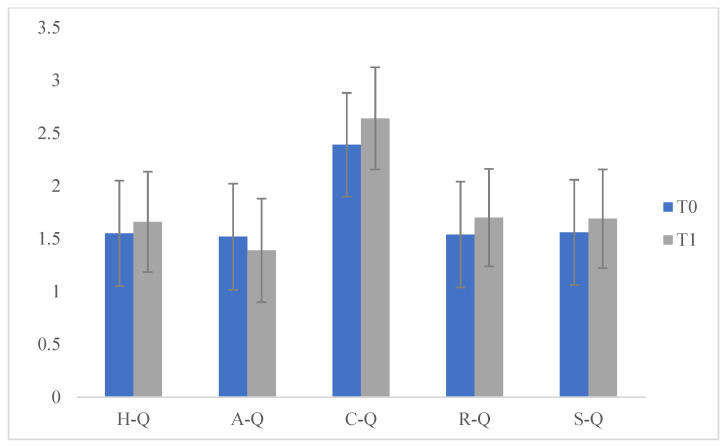
Comparisons of Questionnaire, T0 and T1.

Specifically, we compared the scores which emerged from the VABS administered to the parents at T0 and T1 and significant differences emerged on the (Communication) COM scales (F (1.175) = 3999.877; *p* < 0.05], (socialization) SOC (F (1175) = 34.912; *p* < 0.05), e (personal autonomy) AUT (F (1175) = 72.268; *p* < 0.05) at T2. These data indicate that during the lockdown children experienced improvements in communication, socialization and personal autonomy. We then compared the scores that emerged on the VABS administered to the parents at T1 and T2 and no significant differences emerged. This data indicates that, during the three months of resuming the ABA treatment, there was a maintenance of the previously acquired skills but not a significant improvement. We then compared the scores obtained in the questionnaire (Q) at T0 and T1 with significant differences on the scale (sharing and search of the other) C-Q (F (1175) = 11.578; *p* < 0.05) at T1. This data indicated that during the lockdown the behaviour of "sharing and search for the other" increased and improved; this can be explained by the fact that, during the lockdown period, children received greater environmental stimulation in the family context, which helped them to develop autonomy, increase socialization and communication, as well as fundamental aspects of adaptive and functional development (Table 1 and Figure 1 and Figure 2).

**Figure 3:**
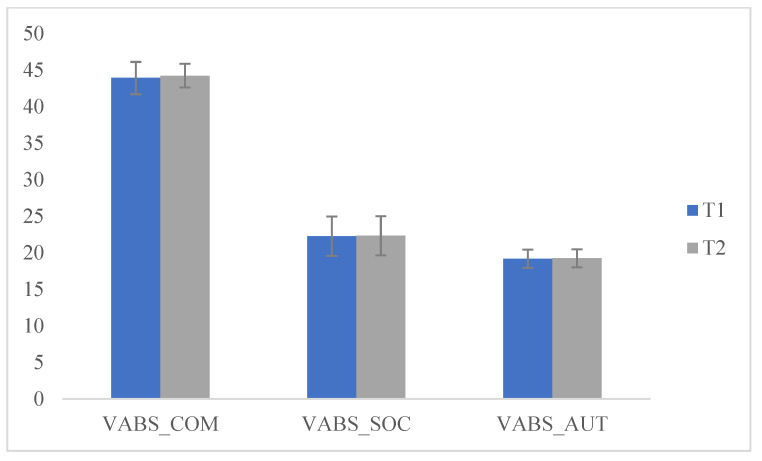
Comparisons VAB, T1 and T2.

**Figure 4:**
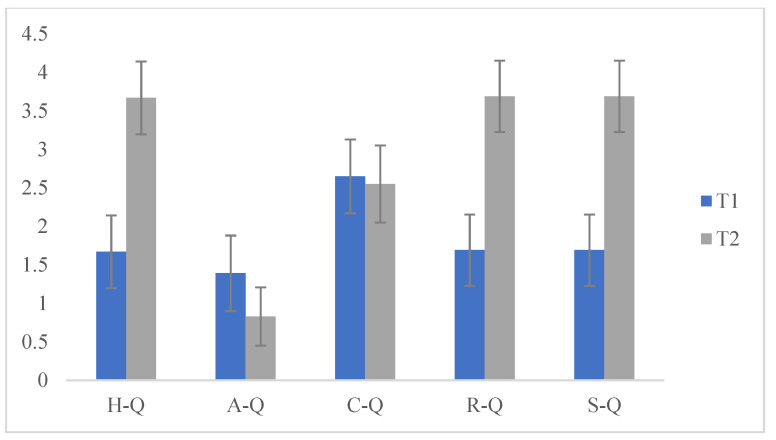
Comparisons Questionnaire, T1 and T2.

Additionally, we compared the scores obtained at T1 and T2 and significant differences emerged on the H-Q (hyperactivity) scale (F (1175) = 787.525; *p* < 0.05), (attention) A-Q (F (1175) = 71.268; *p* < 0.05), (rituals) R-Q (F (1175) = 818.128; *p* < 0.05) and (Stereotypes) S-Q (F (1175) = 818.128; *p* < 0.05). These data indicated that after the lockdown (following the resumption of ABA treatment) there was an increase in hyperactivity and inattention in children, while the sharing research behaviors of the other group remained almost unchanged, highlighted by the non-significance of the scores with respect to T1. Furthermore, there was an increase in stereotypes and ritualization demonstrated by the significance of the scores, since a new change in daily routine caused an increase in problem behaviors (Table 2 and Figure 3 and Figure 4).

## 6. Discussion

The ABA methodology represents a useful tool to work with children affected by Autism Spectrum Disorder. In particular it is useful in increasing socially significant behaviors and decreasing repetitive, stereotyped, problematic, self- and hetero-aggressive behaviors [23]. The effectiveness of this treatment is described in several studies [24,25,26,27,28,29]. The treatment guarantees better results if it is implemented for a longer duration with early intervention; in fact, in children older than two years and affected by autism, when receiving early intensive behavioral intervention better therapeutic results are observed [26,29,30,31,32,33]. In our study we evaluate the impact of the lockdown period (following the quarantine provisions after the outbreak of the COVID-19 pandemic), and the three months following the resumption of activities, in a group of children who underwent ABA treatment. However, many studies report that during treatment, alongside a rapid stabilization of taught behaviors, there may be subsequent difficulty in their maintenance, especially in generalization. In fact, only a genuine involvement of the family and a naturalistic educational context (school) can really favor an effective implementation of the intervention, improving skills in all contexts and making effective the behaviors learnt and proposed during treatment. In our study, the abrupt interruption of treatment, determined by the lockdown, offered us the opportunity to evaluate what the effects of a sudden interruption of an intensive treatment may be. In the observed sample, the parents’ training guaranteed a margin of continuity in the intervention during the six months prior to the lockdown. In particular, in our work we investigated the behavioral aspects of children that undertook ABA treatment for six months. In particular we observed the maintenance of previously acquired skills during the period of suspension of the treatment, and any changes seen in the three months after the resumption of treatment activities. Our analyses revealed a further improvement and a generalization of the skills, rather than a loss, demonstrating the significance of the scores obtained from the administration of the VABS scale at T1 (during the lockdown): communication, socialization and personal autonomy skills improved significantly. At T2 there was a maintenance, but not an increase, in skills, verified through the administration of VABS (three months after the resumption of treatment). An increase in hyperactivity, distractibility, but above all ritualization and stereotypes upon resuming ABA treatment was also observed; in fact, the children encountered difficulties in resuming the activity which had been suspended for about three months. This is evident from the significance of the T2 scores following the administration of the questionnaire to the parents for evaluation of behavioral aspects of their children. Aspects of socialization improved during the lockdown, but remained unchanged when treatment was resumed. With regard to communication, during the lockdown an improvement was observed following the considerable environmental stimuli received in the family context, while skills remained unchanged when the treatment was resumed. The ultimate goal of ABA interventions is to improve and increase communication, knowledge and socially appropriate behaviors (U.S. Department of Health and Human Services) [16], through an effective generalization and maintenance of acquired behaviors. In this study we identified how the drastic interruption of treatment in a group of children (where parents had adequate training) did not cause delays in the generalization of socially significant behaviors. However, upon the resumption of treatment, as demonstrated by the analyses, difficulties occurred, following the change in routines established during the quarantine, making the actual resumption of activities a complex task. In particular, increase in ritualization and stereotypes, highlighted by the significance of the scores, hindered further learning, demonstrating problems of educational control. In fact, while improving their communicative, social and autonomy aspects, children with autism spectrum difficulties maintained a natural tendency towards poor educational control and a propensity for routine and self-referentiality.

## 7. Conclusions

This study highlights the limits of continuity and maintenance of skills acquired through applied behavioral analysis interventions and opens routes to identify more flexible maintenance and generalization procedures in order to improve the integrity of the intervention as a whole. The limitation of this work is that times for studying maintenance and generalization of behaviors related to the ABA treatment proposed by the ASL were dictated by the lockdown, as well as those of the recovery. Therefore, future studies could predict a temporal extension between T0 and T1, and between T1 and T2. Another limitation of the work is the specificity of the sample, referring to a specific age group and a specific level of severity. Future perspectives could consider the possibility of comparing the behavior of different groups by age and level of functioning.

## Figures and Tables

**Table 1:** Comparisons, T0 and T1.

	T0		T1			
	Means	SD	Means	SD	f	*p*
VABS_COM	25.31	1.65	43.92	2.20	3999.877	0.000 *
VABS_SOC	19.24	4.01	22.28	2.69	34.912	0.000 *
VABS_AUT	13.50	6.15	19.19	1.24	72.268	0.000 *
H-Q	1.55	0.500	1.66	0.475	2.286	0.132
A-Q	1.52	0.503	1.39	0.491	2.739	0.100
C-Q	2.39	0.491	2.64	0.483	11.578	0.001 *
R-Q	1.54	0.501	1.70	0.462	4.633	0.033
S-Q	1.56	0.499	1.69	0.467	2.815	0.095

* p < 0.05.

**Table 2:** Comparisons to T1 and T2.

	T1		T2			
	Means	SD	Means	SD	f	*p*
VABS_COM	43.92	2.20	44.23	1.62	1.102	0.295
VABS_SOC	22.28	2.69	22.33	2.67	0.013	0.911
VABS_AUT	19.19	1.24	19.25	1.24	0.091	0.763
H-Q	1.67	0.473	3.67	0.473	787.525	0.000 *
A-Q	1.39	0.490	0.83	0.378	71.268	0.000 *
C-Q	2.65	0.480	2.55	0.501	1.911	0.169
R-Q	1.69	0.464	3.69	0.464	818.128	0.000 *
S-Q	1.69	0.464	3.69	0.464	818.128	0.000 *

* *p* < 0.05.
